# Assessing Therapeutic Alliance in the Context of mHealth Interventions for Mental Health Problems: Development of the Mobile Agnew Relationship Measure (mARM) Questionnaire

**DOI:** 10.2196/jmir.8252

**Published:** 2018-04-19

**Authors:** Katherine Berry, Amy Salter, Rohan Morris, Susannah James, Sandra Bucci

**Affiliations:** ^1^ Division of Psychology and Mental Health School of Health Sciences University of Manchester Manchester United Kingdom

**Keywords:** mobile health, health care provider, digital interventions, therapeutic alliance, mental health, measure development

## Abstract

**Background:**

Digital health interventions in the form of smartphone apps aim to improve mental health and enable people access to support as and when needed without having to face the stigma they may experience in accessing services. If we are to evaluate mobile health (mHealth) apps and advance scientific understanding, we also need tools to help us understand in what ways mHealth interventions are effective or not. The concept of therapeutic alliance, a measure of the quality of the relationship between a health care provider and a service user, is a key factor in explaining the effects of mental health interventions. The Agnew Relationship Measure (ARM) is a well-validated measure of therapeutic alliance in face-to-face therapy.

**Objective:**

This study presented the first attempt to (1) explore service users’ views of the concept of relationship within mHealth mental health interventions and (2) adapt a well-validated face-to-face measure of therapeutic alliance, the Agnew Relationship Measure (ARM), for use with mHealth interventions.

**Methods:**

In stage 1, we interviewed 9 mental health service users about the concept of therapeutic alliance in the context of a digital health intervention and derived key themes from interview transcripts using thematic analysis. In stage 2, we used rating scales and open-ended questions to elicit views from 14 service users and 10 mental health staff about the content and face validity of the scale, which replaced the word “therapist” with the word “app.” In stage 3, we used the findings from stages 1 and 2 to adapt the measure with the support of a decision-making algorithm about which items to drop, retain, or adapt.

**Results:**

Findings suggested that service users do identify relationship concepts when thinking about mHealth interventions, including forming a bond with an app and the ability to be open with an app. However, there were key differences between relationships with health professionals and relationships with apps. For example, apps were not as tailored and responsive to each person’s unique needs. Furthermore, apps were not capable of portraying uniquely human-like qualities such as friendliness, collaboration, and agreement. We made a number of changes to the ARM that included revising 16 items; removing 4 items due to lack of suitable alternatives; and adding 1 item to capture a key theme derived from stage 1 of the study (“The app is like having a member of my care team in my pocket”).

**Conclusions:**

This study introduces the mHealth version of the ARM, the mARM, that has good face and content validity. We encourage researchers to include this easy-to-use tool in digital health intervention studies to gather further data about its psychometric properties and advance our understanding of how therapeutic alliance influences the efficacy of mHealth interventions.

**Trial Registration:**

International Standard Randomized Controlled Trial Number (ISRCTN) 34966555; http://www.isrctn.com/ISRCTN34966555 (Archived by WebCite at http://www.webcitation.org/6ymBVwKif)

## Introduction

Smartphone technology is constantly evolving. Many individuals use their phones and internet regularly, with 89% of adults over the age of 16 owning and using a smartphone [1]. Health care providers are capitalizing on this societal development by exploring innovative ways of using smartphone technology to support the delivery of digital health care interventions (DHIs). Indeed, the National Health Service in the United Kingdom has a digital strategy and mobile technology tool kit that seeks to exploit the capacity of smartphones to improve the efficiency and timeliness of health care interventions [2].

There are now a variety of health-related software applications (apps) available freely to be downloaded for a whole range of health care problems, including mental health problems. The growth in apps for mental health problems is an important development. The near-constant connectivity of smartphones means that people can access support as and when needed without having to overcome the stigma or barriers they might experience in accessing traditional face-to-face mental health services [3]. Accessing face-to-face support can be particularly difficult for those with severe mental health difficulties, such as psychosis, which is characterized by a mistrust of others, impaired social functioning, and difficulties in developing relationships with others [4]. A recent systematic review of Web-based, social media, and mobile technologies for severe mental health problems found that as many as 75% to 95% of service users reported technology-based interventions to be positive and useful for their mental health [5]. Furthermore, DHIs for psychosis have the potential to reduce hospital admissions, improve symptom outcomes, and improve medication adherence [5]. However, research examining the efficacy of mobile health (mHealth) interventions is lagging behind their production. The majority of mHealth interventions are not theory-driven or evidence-based. As with face-to-face delivered interventions, there is an imperative need to evaluate the efficacy of DHIs to ensure that mHealth interventions are actually providing a beneficial treatment.

The concept of therapeutic alliance, a measure of the quality of the relationship between a health care provider and a service user, is a key factor in explaining the effects of face-to-face interventions [6,7]. However, this concept has received little empirical attention in the mHealth field. mHealth interventions present a challenge to the importance of the concepts of alliance and therapeutic relationships, as relationships with health professionals might be diminished, or, in some cases, completely absent. Studies that have investigated the concept of alliance in relation to internet-delivered mental health interventions more generally, including computerized programs, have either assessed therapeutic relationship with a therapist supporting the person to access the technology, or assessed the relationship with the technological device or program itself [8]. These studies have suggested that the concept of alliance may be a less robust predictor of outcomes than in traditional face-to-face interventions for mental health problems, particularly when the direct role of the therapist is minimal and service users are asked to comment on their relationship with a computer or mobile device rather than a therapist assisting with a computer-based intervention (eg, [9-11]). However, there is some evidence that higher scores on these alliance measures are associated with more engagement with interventions [11].

It has been suggested that the relative lack of attention that developers of computerized interventions pay to the relationship-building qualities of self-help technologies for mental health interventions may partly account for the smaller effect sizes compared with face-to-face therapies and higher rates of attrition. For example, the responsivity to data entered, the degree of individually tailored responses, the consistency of advice, and the use of illustrative characters could all enhance the sense of relationship with the device or program in question [8]. There are also problems with how existing studies have measured the concept of alliance within this emerging field. Thus far, existing studies have used measures of alliance developed for use in face-to-face interventions, substituting the word “therapist” with “program.”

In this paper, we described 3 stages of a research process that ultimately aimed to develop a measure of alliance within DHIs. We aimed to build on previous research by exploring service users’ and mental health professionals’ concept of relationship with mHealth interventions and investigated how we might enhance the relationship element of existing mHealth interventions (stage 1). We also used this knowledge from stage 1 of the study, combined with an assessment of the face and content validity of a face-to-face alliance measure (stage 2) to more rigorously adapt the measure for use within the digital health context (stage 3).

## Methods

### Overall Context of the Research

The service user participants from all phases of this study were identified through the Actissist trial [12] (trial registration: ISRCTN34966555), a proof-of-concept trial investigating the feasibility and acceptability of a theory-driven, smartphone-delivered psychological intervention targeting areas of distress in early psychosis. Service users who participated in this research were approached on the basis that they were aged 16 years or older and had been registered under early intervention services across the North West of England, United Kingdom. Consent to participate in this study was obtained through the Actissist trial protocol, which was approved by the relevant ethics and research governance committees. This mixed-methods study occurred across two stages as described below and consisted of 33 participants (n=23 service users; n 10 mental health staff).

### Stage 1: Qualitative Study

Nine service users (7 males, 2 females) participated in one-to-one, face-to-face interviews. A researcher interviewed participants about their views and experience of the Actissist app as well as the concept of therapeutic alliance related to DHIs. Specific questions that pertained most directly to the concept of therapeutic alliance were as follows:

1. Did you feel as though you were in a partnership with the app, like you were working with the app towards a shared goal or purpose?
  Prompt: What are your thoughts about the partnership? Can you tell me more about this experience?
2. Did you think that the app was supportive?
3. Prompt: Why? In what way? Examples?
4. Did you find yourself looking to the app for solutions to your difficulties?
5. Prompt: Can you tell me of an example? How did you feel about looking to an app for solutions?’

Interviews lasted from 45 to 60 min, were recorded, and transcribed verbatim. Interviews were analyzed using thematic analysis [13]. Throughout the analysis, pertinent excerpts of the concept of *relationship* were deductively extracted and labeled with codes. Codes were then organized together to develop themes. The analysis was undertaken by AS under the guidance of KB and SB during regular coding meetings where transcripts were read and coded by all team members to check for consistency in ratings. The organization of the final set of codes into themes was discussed and agreed by the research team.

AS is a postgraduate student studying for a Master’s degree in Clinical and Health Psychology. She has limited direct experience of working with people with mental health problems or in psychological therapies without or without digital components. KB and SB are both clinical psychologists and academics who are experienced in developing therapeutic relationships and carrying out therapy with people with mental health problems. KB has published widely on the concept of therapeutic alliance in people with psychosis. SB has also published on the therapeutic alliance and is the lead investigator on two major trials of DHI for people with psychosis, which includes the Actissist study described in this paper.

### Stage 2: Assessment of Content and Face Validity of the Agnew Relationship Measure

The Agnew Relationship Measure (ARM) is a well-validated measure of therapeutic alliance in face-to-face therapy [14]. The ARM was used as a platform to help develop a measure specifically to assess the concept of alliance in mHealth interventions, which we term the mobile Agnew Relationship Measure (mARM). We chose to adapt the ARM, as this measure has been used in previous studies assessing therapeutic alliance in the context of computerized and mobile-based mental health interventions (e, [9,11,15]). The ARM assesses five concepts thought to comprise therapeutic alliance: bond, partnership, confidence, openness, and client initiative. Bond encompasses feelings of positive regard from and toward the therapist; partnership concerns the collaboration between the client and the therapist; confidence pertains to the competency of the therapist; openness is characterized by the freedom of personal disclosure; and client initiative is associated with feelings of control and empowerment. Consistent with previous studies, the first iteration of the measure that we shared with participants simply replaced the word “therapist” with “app.”

In addition to the ARM, a questionnaire was developed to explore individuals’ opinions about the ARM as a measure for assessing therapeutic alliance in mHealth interventions for psychosis. This included a relevancy scale for each item whereby participants were asked to assess each question on how relevant they believed the item to be to the concept of therapeutic alliance with a DHI on a scale of 1 to 4 (1=not relevant, 2=somewhat relevant, 3=quite relevant, 4=highly relevant). This scale also investigated the wording and format of the questionnaire using open-ended questions that prompted participants to expand on their reasoning or suggest alternative wording of items.

Fourteen service users (none of whom participated in the first part of the study), and 10 members of staff took part in the assessment of the content and face validity of the measure. All participants were presented with the version of the ARM and relevancy scale described above. For convenience, this was done through a range of methods. Eleven participants were interviewed over the phone, 11 were interviewed as part of a small group discussion, and 2 members of staff returned the questionnaire via email. For each method, participants were given at least 24 hours to consider participating in this study. Initially, the participants were contacted over the phone and given information about the study, including the aims of the research. Verbal consent was obtained and a phone interview or face-to-face meeting date was arranged. The relevancy scale (alongside the ARM) was sent to people via email so they were able to familiarize themselves with the measure before the interview.

### Stage 3: Development of the Mobile Agnew Relationship Measure (mARM)

We developed an algorithm for scrutinizing and adapting ARM’s items. The rationale for the algorithm was to support our decision-making process about which items to retain, drop, or reword. The algorithm stated that if at least one service user or staff member rated an item “not relevant” or one participant suggested an alternative wording, then the item was discussed by the research team. For each of these items, an alternative wording was debated, with reference to comments made by staff or service users. Themes and excerpts from stage 1 of the research were also drawn upon in considering the relevance of the item or alternative wording. If the alliance concept of an item did not translate to an mHealth context and no suitable alternative wording could be identified, then the item was removed from the measure.

## Results

### Stage 1: Qualitative Study

Two overarching alliance-related themes were identified from the interviews: (1) forming a bond and relationship with the app; and (2) preference for an app instead of a human therapist. 

**Table 1:** Summary of themes from stage 1 with illustrative quotes.

Theme		Illustrative quote
**Forming a bond and relationship with the app**		
	Building a supportive relationship with the app	“I actually miss doing it cause it does help on different days. Not doing it, I miss it.” [Participant 6]
		“Sometimes, it would have been better for me, I would have preferred to have the app, even now, even though I probably wouldn't use it for 2 or 3 weeks, and still be relying on the information. There comes a time where you mind goes blank to the situation and you're in complete panic mode and to be able to say ‘I need someone. I can't get [Care Coordinator] on the phone, I can't get my care-worker on the phone. What do I do?’” [Participant 1]
	Mimic human support	“It’s another tool in the arsenal...your CPN, he’s only there once a week, there’s a long period of time when you’re on your own, you’ve got nobody. You feel like you’re not wanted, not needed, and that app, it says ‘you’ve got a CPN in your pocket, I’ve got a care provider in my pocket that I can go out quite freely now without my CPN.’” [Participant 1]
		“It did start to feel like part of my normal routine...it was sort of like having a buddy um so yeah, every time it sort of asked you to check in, it was quite a good feeling...it started to seem natural very early...” [Participant 1]
	Barriers to bond	“There’s the reassuring side of I'm not going insane, but theres also the cons to it as well...It’s only a phone telling me I'm not going insane, it's not actually a Dr saying well actually, you’re not that bad.” [Participant 9]
		“I did wonder if it'd be able to portray information as well as a person. Which obviously it can't do because you can't ask it questions.” [Participant 5]
**Preference for an app instead of a human therapist**		
	Apps provide greater freedom than face-to-face	“If you are feeling criticised, you can actually be honest with it...Say, I'm feeling criticised that day and say, be honest with it, it’s - it, yeah, so that’s what’s vital I think. It can create somewhere, instead of having whoever you live with... instead of opening up and having a [an argument], releasing it that way, you can release it into [the app].” [Participant 1]
	Apps promote self exploration	“With the app, you do find yourself opening up a little bit more, personally, on your own privately with your app...Not with your psychologist, not with a CPN, but with yourself and that’s when you find out your being a bit more honest with yourself, you're able to articulate that with your psychologist 'this is how I'm feeling, I know because I've had it on the app'...I've got the app there to be, alone, on my own. I can be an emotional wreck if I want to be, I can be a blubbery, crying baby again if I want to be cons I'm on my own, I'm with my app and it’s getting me through this and that’s what I got from it.” [Participant 1]
		“...You were able to tell it what was worrying you uh - you didn't feel like you couldn't express the worries so um that was good.” [Participant 2]

Themes are summarized in Table 1, along with illustrative quotes supporting the themes generated.

#### Theme 1: Forming a Bond and Relationship With the App

The accounts given by the service users demonstrated that they felt a strong sense of support from the app and consequently, when the app was no longer available for use, people reported missing the support that it had provided. This perceived loss suggested that participants had potentially formed a relational bond with the app. In addition, participants reported that the app provided a sense of security by giving instant support when required.

Several service users indicated that using the app was akin to always having a member of their care team available. Some described it as having a “therapist” in their pocket. Furthermore, a number of participants reported feeling as though they had a friend in the app that would offer encouragement and reassurance. However, there were also accounts where some participants felt that the app was not tailored to their individual needs; the information provided was either repetitive and/or was too generic. This notion was deemed relevant to the concept of developing a relationship with the app, as participants perceived that it was these “robotic” features that hindered the development of a relational bond.

#### Theme 2: Preference for an App Instead of a Human Therapist

In some accounts, participants alluded to a freedom to be honest and open with the app, which was noted as a key difference in the app compared with face-to-face therapy. For example, there seemed to be a reduced risk of embarrassment from confiding in the app as opposed to confiding in a person. This theme was deemed relevant to the concept of relationship, as embarrassment or perceptions of therapist judgment may be a factor in the rupture in face-to-face relationships. Several participants discussed how the app helped them to be honest with themselves and that further down the line, this improved their ability to be open with members of their care team. In this respect, communicating with the app served as a bridge that helped participants to communicate with other people in their social world.

**Table 2:** Service user and staff relevancy rating frequency count. There were 24 respondents overall, but not all respondents provided data for each item.

Modified ARM^a^ items	Not relevant n (%)	Somewhat relevant n (%)	Quite relevant n (%)	Highly relevant n (%)
I feel free to express the things that worry me	1 (5)	0 (0)	9 (45)	10 (50)
I feel friendly towards the app	1 (5)	4 (21)	8 (42)	6 (32)
I am worried about embarrassing myself when using the app	6 (32)	5 (26)	5 (26)	3 (16)
I take the lead when using the app	0 (0)	6 (32)	9 (47)	4 (21)
I keep some important things to myself and don’t share them with the app	5 (26)	2 (11)	5 (26)	7 (37)
I have confidence in the app and its techniques	0 (0)	0 (0)	10 (53)	9 (47)
I feel optimistic about my progress	0 (0)	1 (5)	13 (68)	5 (26)
I feel I can openly express my thoughts and feelings when using the app	0 (0)	2 (11)	8 (42)	9 (47)
I feel critical or disappointed in the app	5 (26)	6 (32)	4 (21)	4 (21)
I can share personal matters I am ordinarily ashamed or afraid to reveal	2 (11)	4 (21)	6 (32)	7 (37)
I look to the app for solutions to my problems	0 (0)	6 (32)	8 (42)	5 (26)
The app’s skills are impressive	1 (5)	8 (42)	8 (42)	2 (11)
The app accepts me no matter how I respond	2 (11)	0 (0)	10 (53)	7 (37)
I feel influenced by the app in ways that are not beneficial to me	6 (32)	3 (16)	6 (32)	4 (21)
The app finds it hard to understand me	7 (37)	3 (16)	5 (26)	4 (21)
The app’s approach is warm and friendly with me	1 (5)	5 (26)	5 (26)	8 (42)
The app does not give me the guidance I would like	5 (26)	4 (21)	4 (21)	6 (32)
The app feels persuasive	4 (21)	7 (36)	5 (26)	3 (16)
The app is supportive	1 (5)	1 (5)	11 (58)	6 (32)
The app follows its own plans, ignoring my views on how to proceed	6 (32)	3 (16)	5 (26)	5 (26)
The app is confident in its messages and techniques	3 (16)	3 (16)	7 (37)	6 (32)
The app seems bored or impatient with me	6 (32)	5 (26)	6 (32)	2 (11)
The app expects me to take responsibility rather than be dependent on it	4 (21)	6 (32)	7 (37)	2 (11)
The app and I are willing to work hard together	3 (16)	6 (32)	8 (42)	1 (11)
I take the lead and the app expects it of me	2 (11)	7 (37)	7 (37)	3 (16)
The app and I agree about how to work together	3 (16)	3 (16)	9 (47)	4 (21)
The app and I have difficulty working jointly as a partnership	5 (26)	6 (32)	6 (32)	2 (11)
The app and I are clear about our role and responsibilities when we interact	2 (11)	6 (32)	8 (42)	3 (16)

^a^ARM: Agnew Relationship Measure.

### Stage 2: Assessment of Face and Content Validity of the Agnew Relationship Measure

Stage 2 of the study aimed to assess the face and content validity of the first iteration of the mARM, which involved simply replacing the word “therapist” with the word “app.” The number of participants who rated each item as not relevant, somewhat relevant, quite relevant, and highly relevant is presented in Table 2. An overarching theme within the relevancy scale and open-ended questions was that some items inappropriately anthropomorphized the app. Both staff and service users reported finding some items hard to answer or relate to the app due to the human nature of the item. For example, the partnership subscales of the ARM, such as agreeing a goal with an app or the concept of the app and the person working together, were particularly criticized. Several staff highlighted the need to qualify items with a perception rather than a statement of fact in instances where human qualities were referenced. For example, replacing phrases, such as “the app is supportive” with “the app seems supportive.” However, staff and service users were more likely to endorse relationship items which reflected their own thoughts and feelings about the app, suggesting that the concept of relationship was important even if it was a less reciprocal relationship. For example, the majority of staff and service users reported that the item, “I feel friendly towards the app” was relevant. Items which referred to techniques proposed by the app or confidence in the app’s capacity to help, which were not uniquely human qualities were also generally less problematic for participants. For example, items such as, “I have confidence in the app and its techniques” or “I feel optimistic about my progress” were endorsed by most people as being relevant. Similarly, items that involved expressing thoughts and feelings, such as “I feel I can openly express my thoughts and feelings when using the app” were felt to be relevant.

### Stage 3: Development of the Mobile Agnew Relationship Measure

Stage 3 of the study aimed to use the results of stages 1 and 2 to reach a consensus about which items to include in the mARM. Eight of the original items were retained, 16 were revised, 4 were removed due to a lack of suitable alternatives and one was added to capture a key theme from the interviews in stage 1 that we felt was not adequately captured by other existing items or other reworded items (“The app is like having a member of my care team in my pocket”). The changes we made to the measure along with the rationale for changing, not changing, or removing items are displayed in Multimedia Appendix 1. The finalized version of the mARM can be found in Multimedia Appendix 2.

## Discussion

### Summary of Findings

This study aimed to explore service users’ views of the concept of *relationship* within mental health DHIs and to examine and adapt a well-validated face-to-face measure of therapeutic alliance for use with mHealth interventions. Stage 1 of this mixed-methods project suggested that service users were able to form a bond and relationship with the app; although, the generic nature of the app’s messages that at times did not feel personalized could hinder the development of this therapeutic relationship. Stage 1 of this study also revealed that some people preferred a relationship with an app as opposed to a relationship with a human therapist. For example, people could be open and honest with the app and this had the potential to promote future communication with other people. Stage 2 of this project suggested that simply replacing the word “therapist” with “the app” in traditional measures of alliance is insufficient to capture the nuances of alliance in mHealth. The findings of stages 1 and 2 were used to inform the development of an mHealth measure of alliance with good content and face validity as determined by both service users and mental health professionals.

### Contextualizing Findings Within Existing Literature

The majority of existing studies have explored alliance in the context of interventions with some degree of overt therapist input into a client’s treatment, such as a therapist assisting the person to use an internet-based self-help program. In these instances, it is difficult to conclude whether existing findings reflected the quality of clients’ working relationships with the therapists involved or with the computer programs themselves. Our study is one of the few studies that have examined the relationship concept in the complete absence of a therapist. Other similar studies have focused on alliance ratings to computer programs for mild to moderate mental health problems [9-11]. These studies suggested that alliance ratings in relation to computer programs were similar to therapist alliance ratings; although, alliance ratings were slightly lower than typical ratings of therapists and alliance was a less important predictor of outcome. However, all of these studies reworded traditional alliance substituting the word “therapist” with “program” (or equivalent term). Future research needs to assess the extent to which the mARM, which was specifically adapted to assess alliance in the mHealth context, is a better predictor of therapy outcomes. However, our finding that some participants felt that the app was not tailored to their individual needs suggested that apps may not yet be sophisticated to mimic human qualities of responsivity and sensitivity to a sufficient degree. At present, truly dialogic engagement may present a challenge for even the most intelligent automated approaches due to its highly contextual and interactive requirements [16].

The findings that people could be more open with the app due to reduced fears about embarrassing themselves compared with talking to a person might suggest that the nonhuman element to the app could prove helpful for some people. In this respect, apps serve a slightly different function to relationships with therapists and consequently perhaps researchers should not be aspiring to fully mimic human interactions when developing a DHI for mental health. Indeed, our adaptions to the ARM items focused on reducing the humanization of the app, which many of the service users and staff in our study found hard to relate to. In making these changes, it was, however, important to hold in mind that we still needed to capture the concept of relationship within our measures rather than developing a measure that purely assesses satisfaction with an app or program.

We are aware that there are existing measures of the quality and functionality of apps [17]. For example, the user Mobile Application Rating Scale ([18]) assesses factors such as an app’s customization, interactivity, ease of use, and the quality and credibility of information. Although the two concepts of quality and alliance are undoubtedly related (eg, if an individual is not satisfied with the quality of the app, it is unlikely that s/he will develop a positive alliance with the app), it is important that measures of alliance in mHealth are sufficiently focused on concepts directly relevant to alliance such as bond, responsiveness to need, and communication, as opposed to other more general indices of app quality.

Our finding that people could be open with the app and empowered by the app reflected Clarke et al’s [11] finding that alliance subscales measuring perceived empowerment and perceived freedom to self-disclose were significantly positively correlated with self-monitoring frequency, suggesting better engagement with the program. Moreover, participants’ ratings of the quality of their emotional connection with the program were positively correlated with program log-ins, frequency of self-monitoring, and number of treatment modules completed. These findings are particularly important as one of the concerns about mHealth interventions is that people are more likely to disengage from them compared with face-to-face therapies [8].

### Limitations

There are some limitations that need to be accounted for when interpreting our findings and considering future uses of the revised mARM. While this research has developed a user-informed measure of alliance for mHealth interventions, the measure requires more rigorous validation. Arguably, this research may not have captured a truly representative range of views about how to measure alliance in mHealth interventions, in particular as participants were recruited from a single clinical population, first episode psychosis.

We attempted to capture the themes derived from stage 1 of the research by rephrasing items in the ARM, using information from the interviews. We also explored an additional item to tap the concept of the app being like a therapist in the pocket. However, arguably the process of basing the measure on the ARM, as opposed to generating items solely from the interviews may have resulted in us missing some potentially important concepts that are uniquely relevant to the assessment of alliance for apps, including the degree to which participants perceive the app’s responses as automated and robotic.

### Summary of Implications

The mARM needs to be subjected to further empirical testing with a wider range of clinical groups. As part of this process, we need to explore whether the mARM is capable of predicting outcomes in DHIs in the same way that therapeutic alliance measures predict outcomes in face-to-face therapy with human therapists. It would also be important to compare the predictive validity of the ARM, which is specifically designed to assess the relationship aspect of therapy with more generic measures of app satisfaction.

### Conclusions

The mARM has attempted to capture unique elements of a digital therapeutic relationship from user feedback. Feedback was obtained from both service users who had first-hand experience with an mHealth intervention and from experienced mental health professionals. The suggestions of these participants and the consequent adaptations we made to an existing, well-established measure of alliance have resulted in an alliance measure with good content and face validity that can assess alliance in mHealth contexts. Simply replacing “therapist” with “the app” in an established measure of alliance is insufficient to capture the nuances of therapeutic alliance in mHealth. The next crucial step in this program of research is to carry out a more comprehensive assessment of the psychometric properties of the scale, ideally within the context of a large mHealth trial, so that the association with outcomes can be systematically explored.

## Appendix

Multimedia Appendix 1 Modification and justification of the amended Agnew Relationship Measure (ARM).

Multimedia Appendix 2 The mARM—the amended Agnew Relationship Measure for use in mHealth interventions.

## Abbreviations

ARM: Agnew Relationship Measure
DHI: digital health interventions
mARM: mobile Agnew Relationship Measure
mHealth: mobile health
