# Supplementary material for: Assessing Therapeutic Alliance in the Context of mHealth Interventions for Mental Health Problems: Development of the Mobile Agnew Relationship Measure (mARM) Questionnaire
Source: J Med Internet Res. 2018 Apr 19;20(4):e90. doi: 10.2196/jmir.8252 (PMC5934536; doi:10.2196/jmir.8252)
Supplement: Multimedia Appendix 1 [file jmir_v20i4e90_app1.pdf]

Multimedia Appendix 1. Modification and justification of the amended Agnew Relationship Measure (ARM).

ARM[a] items	Action	Revised item	Justification
I feel free to express the things that worry me	No action		Only deemed not relevant by one participant, the majority endorsed this item as quite or highly relevant
I feel friendly towards the app	No action		Only deemed not relevant by one participant, the majority endorsed this item as quite or highly relevant
I am worried about embarrassing myself when using the app	Removed		Low relevancy, no alternative option as alternatives suggested were similar to other items
I take the lead when using the app	No action		Not discussed as all participants rated it as relevant to some extent
I keep some important things to myself and don't share them with the app	Revised	I hold back some important things about myself from the app	Low relevancy, reworded to reduce the humanization of the app
I have confidence in the app and its techniques	Revised	I have confidence in the app and the things it suggests	Deemed relevant by all participants but reworded following several alternative suggested wordings
I feel optimistic about my progress	No action		Not discussed as all participants rated it as relevant to some extent
I feel I can openly express my thoughts and feelings when using the app	No action		Not discussed as all participants rated it as relevant to some extent
I feel critical or disappointed in the app	Revised	I feel disappointed in the app	Low relevancy, participants had particular problems with the term "critical."
I can share personal matters I am ordinarily ashamed or afraid to reveal	Revised	I can share personal matters I am normally ashamed or afraid to reveal	Low relevancy, reworded to make it reduce humanization of the app.
I look to the app for solutions to my problems	No action		Not discussed as all participants rated it as relevant to some extent.
The app's skills are	Revised	I have confidence	Low relevancy, reworded to reduce

impressive		in the app and how it works	humanization of the app. One staff member suggested asking about the design/features of the app, but this was felt to move too much away from the concept of alliance.
The app accepts me no matter how I respond	No action		Only deemed not relevant by one participant, and no suitable alternative suggested. The majority endorsed this item as quite or highly relevant.
I feel influenced by the app in ways that are not beneficial to me	Revised	The suggestions the app makes are important to me	Low relevancy, participants highlighted the word “influenced” as being particularly problematic.
The app finds it hard to understand me	Revised	The app seems to understand me	Low relevancy, reworded to reduce the humanization the app. Several staff members recommended using perceptual verbs like seems to.
The app’s approach is warm and friendly with me	Revised	The app’s feels warm and friendly with me	Low relevancy, reworded to reduce the humanization the app. Several staff members recommended using perceptual verbs like seems to.
The app does not give me the guidance I would like	Revised	The app does not give me the help I would like	Low relevancy, reword to reduce the humanization the app. The word “guidance” was highlighted as being particularly problematic.
The app feels persuasive	Removed		Low relevancy, no alternative options were suggested or agreed upon.
The app is supportive	No action		Only deemed not relevant by one participant, the majority endorsed this item as quite or highly relevant.
The app follows its own plans, ignoring my views on how to proceed	Revised	The app seems to ignore my needs	Low relevancy, reworded following the suggestion of one participant.
The app is confident in its messages and techniques	Revised	The app confidently presents its information	Low relevancy. One staff member suggested, “I have confidence and the way it works,” but this was too similar to another item, and it was felt that this item was trying to tap into the messages provided by the app.
The app seems bored	Removed		Low relevancy, no alternative options

or impatient with me			were suggested or agreed upon.
The app expects me to take responsibility rather than be dependent on it	Revised	I am responsible for my recovery, not the app	Low relevancy, reworded to reduce the humanization of the app.
The app and I are willing to work hard together	Revised	The more I use the app, the more I get out of it	Low relevancy, reworded following the suggestion of one participant.
I take the lead and the app expects it of me	Revised	The app gives me the confidence to take the lead in my recovery	Low relevancy, reworded to reduce the humanization of the app.
The app and I agree about how to work together	Revised	I agree with the direction the app is taking me	Low relevancy, reworded to reduce the humanization of the app.
The app and I have difficulty working jointly as a partnership	Removed		Low relevancy, no alternative options were suggested or agreed upon.
The app and I are clear about our role and responsibilities when we interact	Revised	I am clear about what the app can and cannot offer me	Low relevancy, reworded following the suggestion of one participant.

[a]ARM: Agnew Relationship Measure.
