# Supplementary material for: Assessing Therapeutic Alliance in the Context of mHealth Interventions for Mental Health Problems: Development of the Mobile Agnew Relationship Measure (mARM) Questionnaire
Source: J Med Internet Res. 2018 Apr 19;20(4):e90. doi: 10.2196/jmir.8252 (PMC5934536; doi:10.2196/jmir.8252)
Supplement: Multimedia Appendix 2 [file jmir_v20i4e90_app2.pdf]

	Strongly Disagree	Moderately Disagree	Slightly Disagree	Neutral	Slightly Agree	Moderately Agree	Strongly Agree
I feel free to express the things that worry me							
I feel friendly towards the app							
I take the lead when using the app							
I hold back some important things about myself from the app							
I have confidence in the app and the things it suggests							
I feel optimistic about my progress							
I feel I can openly express my thoughts and feelings when using the app							
I feel disappointed in the app							
I can share personal matters I am normally ashamed or afraid to reveal							
I look to the app for solutions to my problems							
I have confidence in the app and how it works							
The app accepts me no matter how I respond							
The suggestions the app makes are important to me							
The app seems to understand me							
The app’s feels warm and friendly with me							
The app does not give me the help I would like							
The app is supportive							
The app seems to ignore my needs							
The app confidently presents its information							
I am responsible for my recovery, not the app							
The more I use the app, the more I							

get out of it							
The app gives me the confidence to take the lead in my recovery							
I agree with the direction the app is taking me							
The app is like having a member of my care team in my pocket							
I am clear about what the app can and can't offer me							
